# Association between pregnancy and severe COVID-19 symptoms in Qatar: A cross-sectional study

**DOI:** 10.1371/journal.pgph.0000891

**Published:** 2023-10-23

**Authors:** Alla’ K. Al-Qassem, Ammar B. Humaidi, Amna K. Al-Kuwari, Elham M. Hasan, Nosaiba H. Yakti, Rakan M. Al-Hathal, Devendra Bansal, Elmoubashar Abu Baker Abd Farag, Hamad E. Al-Romaihi, Mohammed H. J. Al-Thani, Omran A. H. Musa, Suhail Doi, Tawanda Chivese

**Affiliations:** 1 College of Medicine, QU Health, Qatar University, Doha, Qatar; 2 Department of Public Health, Ministry of Public Health, Doha, Qatar; PLOS: Public Library of Science, UNITED STATES

## Abstract

There is inconclusive evidence whether pregnancy exacerbates COVID-19 symptoms or not, and scarce data from the Middle East and North Africa region. The aim of this study was to investigate the association between pregnancy and COVID-19 symptoms in Qatar. This cross-sectional study was carried out using data of all women with confirmed COVID-19, comparing women of child-bearing age (18–49 years). Data of all COVID-19 cases were collected by the Ministry of Public Health (MoPH) in Qatar, between March and September 2020. Symptoms were compared by pregnancy status and classified into moderate and severe. Multivariable logistic and Poisson regression was carried out to investigate the association between pregnancy and severity of COVID-19 symptoms. During the study period, 105 744 individuals were diagnosed with COVID-19, of which 16 908 were women of childbearing age. From that sample, 799 women were pregnant (mean age 29.9 years (SD 5.2)) and 16109 women were not pregnant (mean age 33.1 years (SD 7.8)). After multivariable logistic regression, pregnancy was associated with 1.4-fold higher odds of reporting any symptoms of COVID-19 (OR 1.41, 95% CI 1.18–1.68), and 1.3-fold higher odds of reporting shortness of breath (OR 1.29, 95% CI 1.02–1.63). In a multivariable Poisson regression, pregnancy was also associated with a higher count of symptoms (IRR 1.03, 95%CI 0.98–1.08), although with weak evidence against the null hypothesis. Our findings suggest that, in this setting, pregnant women are more likely to have symptomatic COVID-19, and shortness of breath, compared to women with no pregnancy.

## Introduction

Pregnant women may be at greater risk of acquiring infectious diseases and having severe disease, due to the physiological and immunological changes which occur during pregnancy, that can impair the immune system [1]. Based on this, there are reasonable concerns that COVID-19 could be severe during pregnancy and possibly cause adverse pregnancy outcomes [2]. A severe course of COVID-19 may result in adverse pregnancy outcomes such as stillbirths and maternal deaths [3]. It may therefore be necessary to prioritize protections for pregnant women if they are at higher risk of severe COVID-19.

Although mounting evidence suggests that pregnant women are more susceptible to severe COVID-19, findings on the association between pregnancy and the severity of COVID-19 symptoms are not conclusive. One systematic review and a meta-analysis, with studies up to April 2021, found that pregnant women were at a greater risk of requiring ventilatory support or becoming admitted to an intensive care unit (ICU), in comparison to women of childbearing age with no pregnancy [4]. Another meta-analysis showed an increased risk of severe COVID-19 and a worse symptom profile in pregnant women [5]. In contrast, one systematic review, with studies up to May 2020, found no association between COVID-19 severity, assessed using symptoms and pregnancy. In this review, most pregnant women had mild symptoms, and the clinical presentation of pregnant women was similar to that of the general population [6]. Similarly, another systematic review also reported that certain symptoms such as cough, fatigue, sore throat, headache, and diarrhea to be less probable in pregnant than in women with no pregnancy [7]. The last two reviews demonstrate conflicting findings on the association between pregnancy and COVID-19 symptoms, suggesting a need for more research on this topic. These reviews also did not include studies from the Middle East and North Africa Region. Further, to the best of our knowledge, prior to this study, we found no studies on the association between COVID-19 symptoms and pregnancy in Middle Eastern populations.

There are some suggestions that COVID-19 may become endemic or seasonal, and to have a more benign course due to the high levels of population immunity, because of widespread vaccinations and high proportions of people with prior infection [8]. However, there are still individuals who are at risk of severe symptomatic COVID-19, either because of comorbidities, advanced age or due to the development of new variants of the disease [9]. Certain symptoms such as shortness of breath have been shown to be prognostic for severe COVID-19. For instance, one study showed that shortness of breath was associated with 2.4-folds odds of severe COVID-19 [10]. Other symptoms have also been shown to be associated with severe COVID-19 although the data are inconclusive [11]. Further, it is likely that a higher symptoms count could imply a worse disease profile, however, there is little research on this.

This study investigated the association between pregnancy and COVID-19 symptoms in a Middle Eastern population where such data have been scarce to date. The primary objective of this study was to compare COVID-19 symptomatic status between women with and without pregnancy. We also compared individual symptoms between groups of women with and without pregnancy, as well as within age groups.

## Materials and methods

### Design and setting

This study was a cross-sectional study of women of childbearing age with confirmed COVID-19 using data collected by the Ministry of Public Health (MoPH) in Qatar during the period of March to September 2020. During the study period, the MoPH collected data on all confirmed cases of COVID-19, irrespective of disease severity. COVID-19 was diagnosed by reverse transcriptase polymerase chain reaction (RT-PCR), and screening was done through several ways, including mandatory contact tracing and testing of all arrivals into the country and individuals with symptoms. Symptomatic status was evaluated across two groups, women with or without pregnancy. Additionally, in both groups individual symptoms were compared and categorized into mild to moderate and severe classes of symptoms according to a modified version of the criteria proposed by the National Institutes of Health (NIH) of the United States [12]. The study is reported following the Strengthening the Reporting of Observational Studies in Epidemiology (STROBE) (S1 Text).

### Study participants

The population of Qatar consists of a large heterogeneous multinational population of which expatriates form the majority, around 90%, and the remainder are local [13]. This population was also reflected in the confirmed cases of COVID-19 from the MoPH. For this study, participants were eligible if they were women aged between 18 to 49 years of age and if they had confirmed COVID-19 by RT-PCR. We excluded individuals below 18 years or above 49 years of age and women in the postpartum period, defined as within 6 weeks after birth.

### Data collection

From the MoPH dataset, we selected data for each participant which included symptoms of COVID-19, pregnancy status, sociodemographic data such as age, nationality, marital status, month of diagnosis, number of symptoms and comorbidities. The data on symptoms were collected by telephonic interview by the MoPH. Each individual was asked about presence of respiratory symptoms, such as cough, sore throat, and shortness of breath, gastrointestinal symptoms, such as diarrhea and abdominal pain, musculoskeletal symptoms that included muscle or joint pain, and systemic symptoms which consisted of fever, headache, and chills. The symptoms were classified into two categories, one for mild to moderate, which included fever, cough, sore throat, headache, muscle or joint pain, diarrhea, chills, or abdominal pain, but not shortness of breath, and another for severe symptoms, which was made up of shortness of breath. The severity categories were then compared between women without pregnancy and with pregnancy. We also compared individual symptoms by pregnancy status, and, further, compared both severity categories and individual symptoms by pregnancy status but within the age groups of 18–29 years, 30–39 years, and 40–49 years of age. Further, we categorized individual symptoms into organ systems and compared frequency across the three age groups.

### Statistical analysis

The categorical data were summarized as frequencies and percentages, whereas age was normally distributed and therefore summarized using mean and standard deviation (SD). To compare the categorical data by pregnancy status, the chi-squared (χ2) test was utilized, and the t-test was used to compare mean age. We utilized literature and directed acyclic graphs (S1 Fig) to identify potential confounders to adjust for in the association between pregnancy and COVID-19, and in this case, only age appeared to meet the confounding criteria [14–16]. We also adjusted for diabetes, cardiovascular disease (CVD), and region of origin as these have been shown to be prognostic for severe COVID-19 [17, 18]. We compared individual symptoms and symptom categories using the chi-squared test. Multivariable logistic regression was then carried out to assess the association between pregnancy and the severity of COVID-19 symptoms, adjusted for age, diabetes, CVD, and region of origin (with Asia as the reference). We carried out two models, the first model with the outcome being any symptomatic COVID-19 compared to no symptoms, the second model with the outcome being shortness of breath compared to no symptoms. The odds ratios (OR), 95% confidence intervals (95% CI), and exact p-values were reported. We tested model specification using the link test and model fit using the area under the receiver operating curve (ROC curve). To control for the effect of age, we carried out a stepwise logistic model with COVID-19 symptoms (yes/no) as the outcome, but age was dropped from this model and therefore not considered in the final analysis. Further, we carried out multivariable Poisson regression with the outcome being the count of symptoms and the exposure being pregnancy and adjusted for the same confounders. All analyses were carried out in Stata version 16 [19].

### Ethics approval

Ethics approval and waiver of informed consent were approved by the MoPH (ERC-826-3-2020), and all data were de-identified before the analysis.

## Results

### Characteristics of included participants

**Fig 1:**
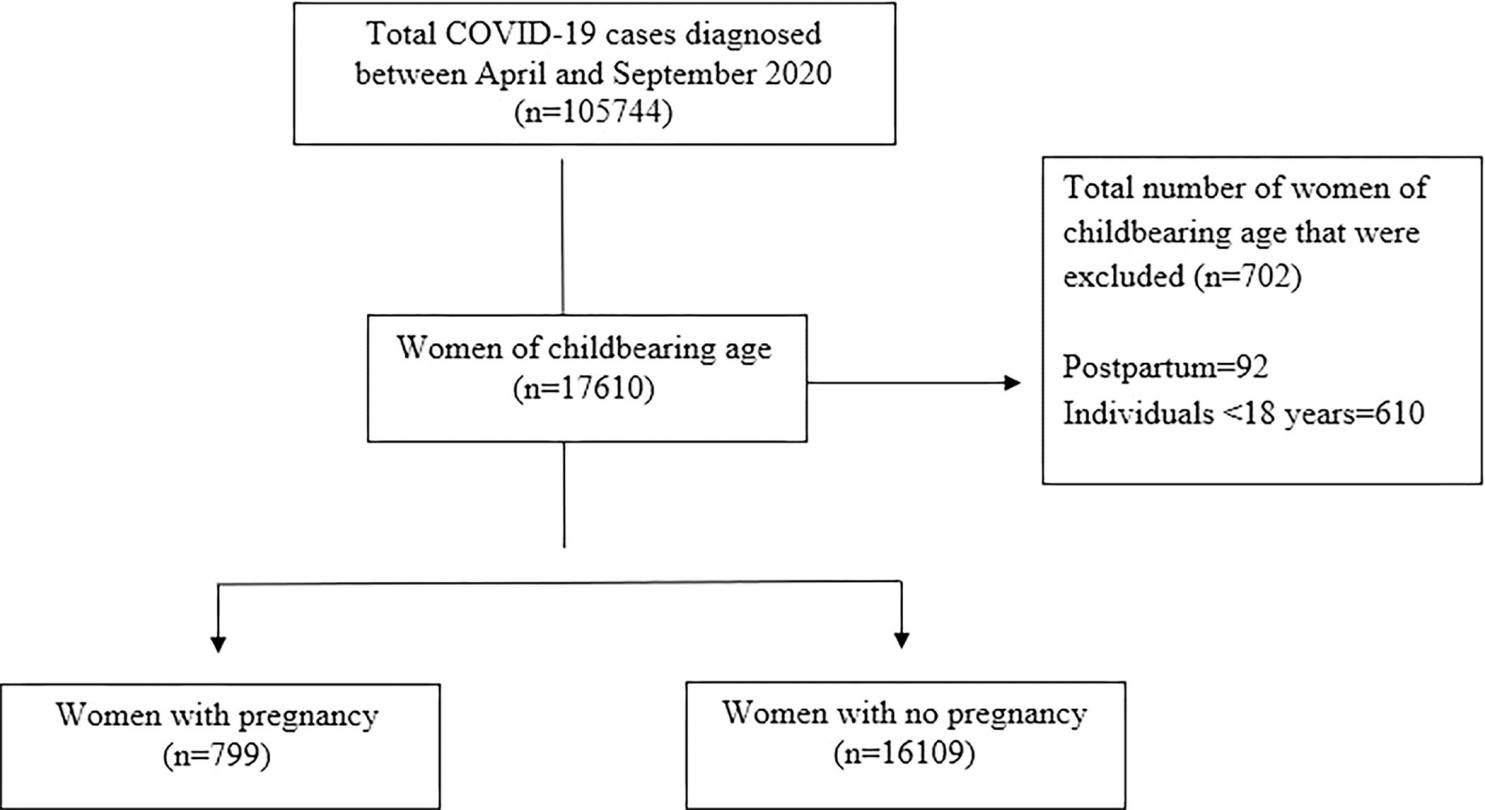
Study flow chart. Figure showing the flow of participants in the study.

A total of 105 744 individuals were diagnosed with COVID-19 during the study period. From these, 17 610 were women of childbearing age. After exclusion of women in post-partum and those who were aged less than 18 years, 799 pregnant and 16 109 women without pregnancy were included (Fig 1). The characteristics of the included participants, by pregnancy status, are shown in Table 1.

**Table 1:** Characteristics of participants by pregnancy status.

Variable		Overall, n = 16908	Pregnant, n = 799	No pregnancy, n = 16109	P-value
Age, years	Mean (SD)	33.0 (7.7)	29.9 (5.2)	33.1 (7.8)	<0.001
Age groups, n (%)					<0.001
	18–29 years	5929 (35.1)	380 (47.6)	5549 (34.4)	
	30–39 years	7224 (42.7)	393 (49.2)	6831 (42.4)	
	40–49 years	3755 (22.2)	26 (3.2)	3729 (23.1)	
Region of origin, n (%)					<0.001
	Asia	8105 (47.9)	290 (36.3)	7815 (48.5)	
	Middle east & north Africa	7746 (45.8)	477 (59.7)	7269 (45.1)	
	Sub-Saharan Africa	767 (4.5)	22 (2.8)	745 (4.6)	
	North America & Europe	255 (1.5)	8 (1.0)	247 (1.5)	
	Australasia	13 (0.1)	0 (0.0)	13 (0.1)	
	South America	21 (0.1)	2 (0.2)	19 (0.1)	
Marital status, n (%)					<0.001
	Married	5917 (35.0)	487 (60.9)	5427 (33.7)	
	Single	1763 (10.4)	5 (0.6)	1758 (10.9)	
	Divorced/widowed	77 (0.5)	0 (0.0)	9 (0.1)	
	Not applicable	9144 (54.1)	295 (36.9)	2069 (12.8)	
	Others	9 (0.1)	0 (0.0)	9 (0.1)	
Month of diagnosis, n (%)					<0.001
	April	546 (3.2)	35 (4.4)	511 (3.2)	
	May	2816 (16.7)	174 (21.8)	2642 (16.4)	
	June	4780 (28.3)	202 (25.3)	4578 (28.4)	
	July	2468 (14.6)	128 (16.0)	2340 (14.5)	
	August	3244 (19.2)	139 (17.4)	3105 (19.3)	
	September	3054 (18.1)	121 (15.1)	2933 (18.2)	
Number of symptoms	Medium (IQR)	2 (0–3)	2 (0–3)	2 (0–3)	0.0138
Comorbidities, n(%)					
	Cardiovascular Disease	537 (3.2)	12 (1.5)	525 (3.3)	0.006
	Diabetes	601 (3.6)	60 (7.5)	541 (3.4)	<0.001
Death, n (%)		3 (0.02)	0 (0.0)	3 (0.02)	

*Column percentages are reported

*Values are numbers (percentages) unless stated otherwise.

Table with demographic information of participants in the study by pregnancy status. Column percentages are reported, and values are numbers (percentages) unless stated otherwise.

The mean age of the included women was 33.0 years (SD 7.7), with most of the individuals being aged between 30–39 years (42.7%) and 18–29 years (35.1%) (Table 1). Pregnant women were slightly younger than those without pregnancy (mean 29.9 years (SD 5.2) vs 33.1 years (SD 7.8), respectively, p<0.01). Most of the women were from countries in the Asia (47.9%), followed by countries in Middle East & North Africa (45.8%). Most of the participants were diagnosed in the months of June and August (28.3% and 19.2%, respectively). There were three deaths (0.02%), all in women with no pregnancy during the study period. The first case was of a 43-year-old lady with diabetes who had dyspnea, cough, and fever. The second case was of a 48-year-old with no comorbidities who had fever alone. The third case was of a 25-year-old with no comorbidities who had fever only.

### Comparison of symptoms between women with and without pregnancy

Table 2 compares COVID-19 symptoms between pregnant women and women with no pregnancy. Overall, the proportion of symptomatic COVID-19 was higher in the pregnancy group compared to the group of women without pregnancy (78.3% vs 70.5%, respectively, p<0.001), with strong evidence against the null hypothesis. In terms of individual symptoms, compared to women without pregnancy, a significantly higher proportion of pregnant women reported shortness of breath (10.6% vs 7.9%, respectively, p = 0.006) and cough (37.3% vs 32.9%, p = 0.016). No significant differences were observed between women with and without pregnancy across the other symptoms.

**Table 2:** Comparison of severity of symptoms between by pregnancy status.

Variable, n(%)	Pregnant women, n = 799	No pregnancy, n = 16109	P value
Asymptomatic, n = 4920	173(21.7)	4747(29.5)	<0.001
Symptomatic, n = 11988	626(78.3)	11362(70.5)	<0.001
Mild to moderate symptoms, n = 10628			
Fever	301(42.2)	6078(41.0)	0.53
Cough	266(37.3)	4882(32.9)	0.016
Sore throat	171(23.9)	3587(24.2)	0.89
Headache	234(32.8)	4644(31.3)	0.41
Muscle or joint pain	211(29.6)	3905(26.3)	0.056
Diarrhoea	31(4.3)	855(5.8)	0.11
Chills	57(8.0)	1069(7.2)	0.43
Abdominal pain	49(6.9)	842(5.7)	0.18
Severe group n = 1360			
Shortness of breath	85(10.6)	1275(7.9)	0.006

Table comparing COVID-19 symptoms between pregnant women and women with no pregnancy. The symptoms are classified into two categories, mild to moderate, which included fever, cough, sore throat, headache, muscle or joint pain, diarrhea, chills, or abdominal pain, but not shortness of breath, and severe symptoms, which was made up of shortness of breath.

### Comparison of symptoms by pregnancy status within age groups

Table 3 shows a comparison of COVID-19 symptoms by pregnancy across different age categories. Overall, the proportion of symptomatic COVID-19 was significantly higher in pregnant compared to women with no pregnancy in the age groups of 18–29 years (76.5% vs 69.6%, respectively, p<0.001), and 30–39 years (78.1% vs 70.8%, respectively, p = 0.002). Shortness of breath was also reported by a higher proportion of pregnant women compared to women with no pregnancy in the age group of 18–29 years (13.2% vs 7.4%, respectively, p<0.001). With regards to mild to moderate symptoms, most symptoms were similar across both groups in the age categories, with a few exceptions. These included cough (38.2% vs 32.7%, respectively, p<0.040) and muscle or joint pain (29.7% vs 24.3%, respectively, p = 0.029) which were greater in pregnant women compared to women without pregnancy in the age group of 18–29 years, respectively. Additionally, sore throat was more common in pregnant women than women without pregnancy in the age group 30–39 years (28.3% vs 23.3%, respectively, p = 0.030). However, in the age group of 18–29 years, sore throat was of greater proportion in women without pregnancy compared to pregnant women in the age group of 18–29 years (25.7% vs 19.1%, respectively, p = 0.008), Additionally, fever was more frequent in women with no pregnancy than it was in pregnant women in the age group of 40–49 years (41.4% vs 17.4%, respectively, p = 0.020). The COVID-19 symptoms were further combined and categorized based on the organ system (S1 Table). In general, respiratory symptoms were significantly higher in pregnant versus women with no pregnancy in the age group of 18–29 years (52.9% vs 47.7%, respectively, p = 0.050), and 30–39 years (52.9% vs 47.1%, respectively, p = 0.023). In addition, musculoskeletal symptoms were more common in pregnant women than women with no pregnancy in age group 18–29 years (33.2% vs 26.8%, respectively, p = 0.007).

**Table 3:** Comparison of severity of symptoms by pregnancy status in each age group.

Variables	Pregnant, 18–29 years (n = 380)	No pregnancy, 18–29 years (n = 5549)	*P* value	Pregnant, 30–39 years (n = 393)	No pregnancy 30–39 years (n = 6831)	*P* value	Pregnant, 40–49 years (n = 26)	No pregnancy, 40–49 years (n = 3729)	*P* value
Asymptomatic n = 4920	78(20.5)	1689(30.4)	<0.001	86(21.9)	1993(29.2)	0.002	9(34.6)	1065 (28.6)	0.50
Symptomatic n = 11988	302(76.5)	3860(69.6)	<0.001	307(78.1)	4838(70.8)	0.002	17 (65.4)	2664 (71.4)	0.50
Mild to moderate symptoms, n = 10628									
Fever	144(43.6)	2128(41.4)	0.42	153(42.4)	2540(40.8)	0.46	4(17.4)	1410(41.4)	0.020
Cough	126(38.2)	1681(32.7)	0.040	134(37.1)	2028(32.3)	0.056	6(26.1)	1173(34.4)	0.40
Sore throat	63(19.1)	1319(25.7)	0.008	102(28.3)	1462(23.3)	0.030	6(26.1)	806(23.6)	0.78
Headache	99(30.0)	1668(32.5)	0.36	130(36.0)	1959(31.2)	0.054	5(21.7)	1017(29.8)	0.40
Muscle or joint pain	98(29.7)	1251(24.3)	0.029	110(30.5)	1710(27.2)	0.18	3(13.0)	944(27.7)	0.12
Diarrhea	15(4.5)	287(5.6)	0.42	16(4.4)	373(5.9)	0.24	0(0.0)	195 (5.7)	0.24
Chills	31(9.4)	347(6.8)	0.067	24(6.6)	466(7.4)	0.59	2(8.7)	256 (7.5)	0.83
Abdominal pain	25(7.6)	300(5.8)	0.20	24(6.6)	345(5.5)	0.35	0(0.0)	197(5.8)	0.24
Severe symptoms, n = 1360									
Shortness of breath	50(13.2)	409(7.4)	<0.001	32(8.1)	546(8.0)	0.92	3(11.5)	320 (8.6)	0.59

Table comparing COVID-19 symptoms between pregnant women and women with no pregnancy, within the age groups of 18–29 years, 30–39 years, and 40–49 years of age.

### Association between pregnancy and symptomatic COVID-19

After multivariable logistic regression, pregnancy was associated with higher odds of symptomatic COVID-19 (OR 1.41, 95% CI 1.18–1.68, p<0.01). Further, pregnancy was associated with higher odds of shortness of breath (OR 1.29, 95% CI 1.02–1.63, p = 0.03) (Table 4). In stepwise logistic regression models, with p = 0.3 for removing a variable from the model, pregnancy, diabetes, CVD and region of origin were potential predictors of both COVID-19 symptom status and shortness of breath in women of childbearing age, but age was dropped from the models (S2 Table). There was also no association with age of the women of childbearing age when age was categorized to less than and at least 35 years of age (S2 Table). This lake of association between age and COVID-19 symptoms was most likely because the women in this study were mostly in the narrow age range of 18–39 years, especially those who were pregnant. Therefore, the effect of age observed in other studies with participants without pregnancy was not observed.

**Table 4:** Association between pregnancy and symptomatic COVID-19 –multivariable logistic regression.

Variable	Symptomatic#1			Shortness of breath #2		
	OR	P-value	95% CI	OR	P-value	95% CI
Pregnancy	1.41	0.00	1.18–1.68	1.29	0.03	1.02–1.63
Age	1.00	0.17	1.00–1.01	1.01	0.12	1.00–1.01
CVD	1.89	0.00	1.49–2.38	1.94	0.00	1.48–2.50
Diabetes	1.72	0.00	1.37–2.15	1.44	0.01	1.12–1.86
Region of Origin						
Asian	Base					
Middle east & north Africa	1.62	0.00	1.51–1.74	1.79	0.00	1.59–2.01
South America	0.45	0.07	0.19–1.06	1.63	0.51	0.38–7.01
Sub-Saharan Africa	0.53	0.00	0.46–0.62	0.60	0.01	0.41–0.89
Australasia	5.61	0.10	0.73–43.27	2.48	0.24	0.54–11.32
North America & Europe	1.65	0.00	1.23–2.22	0.75	0.33	0.42–1.35

A multivariable logistic regression table with two models:

#1 –A singles multivariable logistic regression model with the outcome of symptomatic COVID-19 (yes/no). Model statistics: Number of observations = 16 907, LR Chi = 429.03, P-value = 0.0000, Pseudo R2 = 0.021, hatsq (from linktest) = 0.162, area under the ROC curve = 0.60

#2 –A singles multivariable logistic regression model with the outcome of shortness of breath (yes/no). Model statistics: Number of observations = 16 907, LR Chi = 179.16, P-value = 0.0000, Pseudo R2 = 0.024, hatsq (from linktest) = 0.710, area under the ROC curve = 0.62

### Association between pregnancy and the number of COVID-19 symptoms

After multivariable Poisson regression, pregnancy was associated with a higher count of symptoms (IRR 1.03, 95% CI 0.98–1.08, p = 0.31), although with weak evidence against the null hypothesis (Table 5).

**Table 5:** Association between the count of symptoms and pregnancy–multivariable Poisson regression.

	Number of symptoms		
	IRR	P-value	95% CI
Pregnancy	1.03	0.31	0.98–1.08
Age (overall)	1.00	0.16	1.00–1.00
CVD	1.24	0.00	1.17–1.31
Diabetes	1.19	0.00	1.13–1.25
Region of origin			
Asia	Base		
Middle east & north Africa	1.47	0.00	1.44–1.50
South America	0.93	0.67	0.66–1.31
Sub-Saharan Africa	0.68	0.00	0.64–0.74
Australasia	1.76	0.00	1.28–2.41
North America & Europe	1.12	0.01	1.02–1.23

A multivariable Poisson regression table with the outcome being the number of symptoms and the exposure being pregnancy having adjusted for the same confounders.

## Discussion

In this population based cross sectional study, we found that, in a diverse Middle Eastern population, pregnant women were more likely to report symptomatic COVID-19 compared to women with no pregnancy. Moreover, pregnant women had a higher proportion of shortness of breath, compared to women who were not pregnant. In multivariable logistic regressions, pregnancy was associated with 1.41-fold higher odds of having symptomatic COVID-19 and almost 1.3-fold higher odds of reporting shortness of breath. Moreover, a multivariable Poisson regression analysis showed that pregnant women were more likely to have a higher number of symptoms compared to women with no pregnancy.

We found higher odds of symptomatic COVID-19 and shortness of breath in pregnant women compared to women with no pregnancy of the same age. Although there are some contrasting results from other studies [7, 20, 21], our findings add to a growing body of evidence that suggests that pregnant women are vulnerable to severe COVID-19 [4, 5, 22, 23], albeit from a region where such data have been scarce to date. In one meta-analysis of 67 271 women, pregnant women and women in the postpartum group were twice more likely to be admitted to ICU, to have mechanical ventilation and to require extracorporeal membrane oxygen [4]. In another study from the US CDC, of 461 825 women, pregnant women three times more likely to be admitted to ICU, two-and-a-half times to receive extracorporeal membrane oxygenation, three times more likely to need mechanical ventilation and almost twice likely to die from COVID-19. In contrast, one systematic review concluded that clinical features of pregnant women with COVID-19 did not vary from those of women with no pregnancy [20]. However, this review had several limitations including a very small sample size of 114 total participants and included poor-quality studies. Similarly, two other older reviews suggested that there was no difference in COVID-19 presentation between women with and without pregnancy [21], and that pregnant women were less likely to have cough, sore throat, headache and diarrhea than those were not pregnant [7]. Notably, these older reviews included small and poorly designed studies. Taken together, our findings and those of existing studies suggest a worse course of symptoms of COVID-19 in pregnancy.

In multivariable logistic regression models, age, in its continuous form or categorized, did not seem to affect the observed associations between pregnancy and COVID-19 symptoms. Data on the association between COVID-19 symptoms and pregnancy in different age groups remains scarce. One cross sectional study in Saudi Arabia reported that women with advanced maternal age were more likely to be symptomatic, with a greater acute respiratory illness median score [24]. However, the study did not include women without pregnancy, thus, more research is needed.

Several physiological changes during pregnancy may worsen the course of COVID-19 and therefore result in severe symptoms. A more symptomatic course of the disease could be caused by changes in the respiratory system during pregnancy that can impair the protective functions of the lungs [25]. Also, high levels of estrogen and progesterone hormones during pregnancy induce the upper part of the respiratory tract to swell, which increases the susceptibility of severe COVID-19 infection. Lastly, pregnancy is a hypercoagulable state, and this makes pregnant women more vulnerable to a severe course of COVID-19, where coagulation dysfunction is a hallmark of poor disease outcomes [26, 27].

There are several limitations in our study. Firstly, data collection from participants was done through phone calls, to reduce the spread of COVID-19 at those early times in the pandemic. However, there is no basis to believe that this subjective reporting of symptoms was differential, such that it was more likely in either pregnant women or women with no pregnancy. Therefore, if this subjective assessment affected the study, it would most likely have biased the odds ratios towards the null, and the expected effect if objective assessment was done, would probably show higher odds ratios. Additionally, information about the gestational age of the participants was not measured, which may have affected the symptomology of COVID-19, as immunological and physiological changes vary throughout between pregnancy trimesters. Further, dyspnea is a predominant clinical presentation in severe COVID-19, but also in most pregnancies during their third trimester [28]. Further studies investigating the effect of COVID-19 infection on pregnancy in different trimesters are therefore needed. There is a chance that pregnant women were more likely to do the PCR test, compared with women without pregnancy, as they were in contact with the healthcare system at the time. However, this study compared symptoms in the women who had PCR tests by pregnancy status, and therefore would not have been affected by a higher propensity of pregnant women to get PCRs. It is also likely that women who were pregnant were more likely to monitor their symptoms, and report them, compared to women without pregnancy, especially when not severe. This may have affected the findings of the study and resulted in higher odds of symptom reporting in pregnancy. An important question which we were not able to answer in this research is the effect of COVID-19 on pregnancy outcomes, and future research may be needed in this setting. Despite this, this study allowed us to explore the symptoms of COVID-19 infection across a multicultural cohort, using a large sample with a diversity of participants.

## Conclusion

Our findings suggest that, in a multinational cohort in Qatar, pregnant women are more likely to have symptomatic COVID-19, especially shortness of breath, compared to women without pregnancy of the same age. Policies are required to ensure that women who are pregnant receive more follow ups during public health emergencies such as COVID-19, while limiting unnecessary contact with the healthcare personnel. Telehealth could be an option, which can be used to monitor the severe symptoms in pregnant women. Another consideration is the need to explore interventions that can help in treating symptoms and preventing deterioration in pregnant women once they present with COVID-19. Finally prioritizing women who are pregnant for preventive measures such as vaccinations may help in reducing unnecessary morbidity from COVID-19 infection in women who are pregnant.

## Supporting information

S1 Text STROBE checklist. Checklist showing the Strengthening the Reporting of Observational Studies in Epidemiology (STROBE).
(DOCX)

S1 Fig DAG for confounders of pregnancy and COVID-19 symptoms. Directed acyclic graphs (DAG) to identify potential confounders to adjust for in the association between pregnancy and COVID-19, and in this case, only age was needed to control for confounding.
(TIF)

S1 Table Comparison of organ systems-based symptoms by pregnancy status in each age group. A table comparing organ-based systems, including respiratory, gastrointestinal, musculoskeletal, and systemic systems by pregnancy status in each age group.
(DOCX)

S2 Table Stepwise logistic regression for association between symptomatic COVID-19 and pregnancy. A Stepwise logistic regression model with COVID-19 symptoms (yes/no) as the outcome.
(DOCX)
