# Supplementary figures and images for: Association between pregnancy and severe COVID-19 symptoms in Qatar: A cross-sectional study
Source: PLOS Glob Public Health. 2023 Oct 23;3(10):e0000891. doi: 10.1371/journal.pgph.0000891 (PMC10593215; doi:10.1371/journal.pgph.0000891)

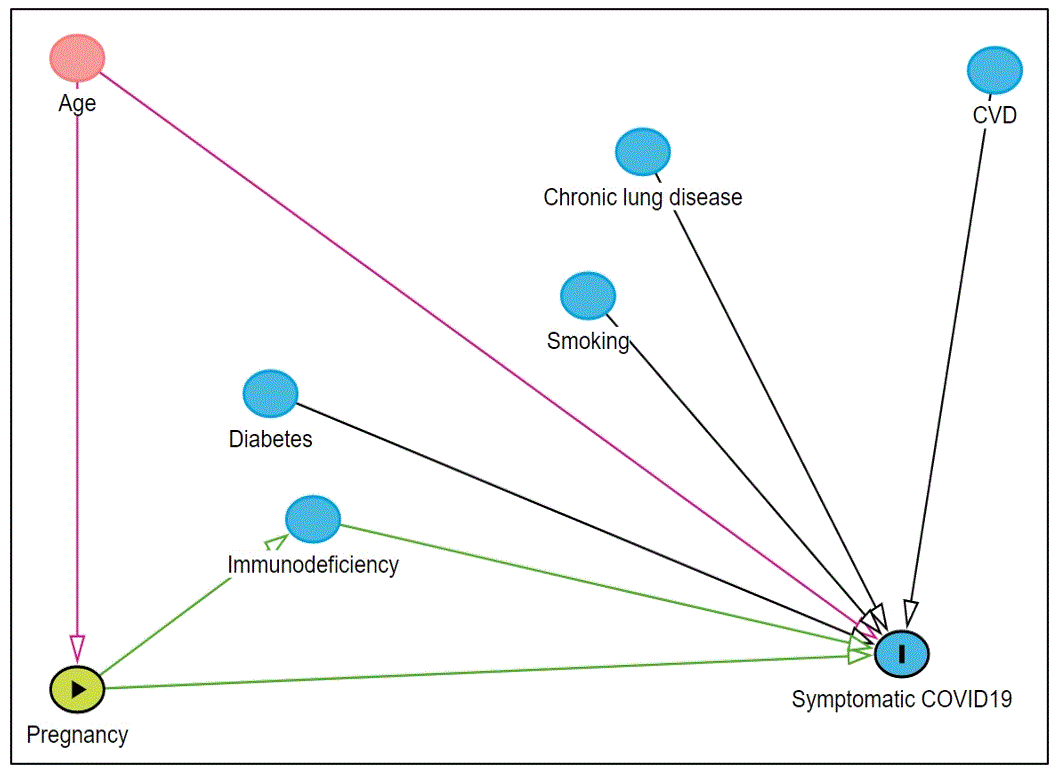

Supplement: S1 Fig — Directed acyclic graphs (DAG) to identify potential confounders to adjust for in the association between pregnancy and COVID-19, and in this case, only age was needed to control for confounding. (TIF) [file pgph.0000891.s002.tif]
