# Supplementary material for: Association between pregnancy and severe COVID-19 symptoms in Qatar: A cross-sectional study
Source: PLOS Glob Public Health. 2023 Oct 23;3(10):e0000891. doi: 10.1371/journal.pgph.0000891 (PMC10593215; doi:10.1371/journal.pgph.0000891)
Supplement: S1 Table — A table comparing organ-based systems, including respiratory, gastrointestinal, musculoskeletal, and systemic systems by pregnancy status in each age group. (DOCX) [file pgph.0000891.s003.docx]

**S1 Table: Comparison of organ systems-based symptoms by pregnancy status in each age group**

| Variables | Pregnant  18-29 years (n%) | No pregnancy 18-29 years (n%) | P value | Pregnant  30-39 years (n%) | No pregnancy 30-39 years (n%) | P value | Pregnant  40-49 years (n%) | No pregnancy 40-49 years (n%) | P value |
| --- | --- | --- | --- | --- | --- | --- | --- | --- | --- |
| Respiratory symptoms n% | 201(52.9) | 2647(47.7) | 0.050 | 208(52.9) | 3214(47.1) | 0.023 | 12(46.15) | 1824(48.9) | 0.779 |
| Gastrointestinal symptoms n% | 48(12.6) | 613(11.1) | 0.342 | 42(10.7) | 740(10.8) | 0.928 | 2(7.69) | 393(10.54) | 0.637 |
| Musculoskeletal symptoms n% | 126(33.2) | 1488(26.8) | 0.007 | 130(33.1) | 2020(29.6) | 0.139 | 5(19.2) | 1115(29.9) | 0.236 |
| Systemic symptoms n% | 231(60.8) | 3191(57.5) | 0.210 | 235(59.8) | 3809(55.8) | 0.117 | 11(42.3) | 2110(56.6) | 0.143 |
