# Supplementary material for: Association between pregnancy and severe COVID-19 symptoms in Qatar: A cross-sectional study
Source: PLOS Glob Public Health. 2023 Oct 23;3(10):e0000891. doi: 10.1371/journal.pgph.0000891 (PMC10593215; doi:10.1371/journal.pgph.0000891)
Supplement: S2 Table — A Stepwise logistic regression model with COVID-19 symptoms (yes/no) as the outcome. (DOCX) [file pgph.0000891.s004.docx]

**S2 Table: Stepwise logistic regression for association between symptomatic COVID-19 and Pregnancy**

| Variables | Symptomatic | | | Shortness of breath | | |
| --- | --- | --- | --- | --- | --- | --- |
|  | OR | P-value | 95% CI | OR | P-value | 95% CI |
| Pregnancy | 1.39 | 0.00 | 1.16-1.65 | 1.22 | 0.15 | 0.93-1.62 |
| Age ≥ 35 years | 0.98 | 0.53 | 0.91-1.05 | 0.99 | 0.88 | 0.86-1.14 |
| Diabetes | 1.76 | 0.00 | 1.41-2.20 | 1.40 | 0.03 | 1.03-1.88 |
| CVD | 1.94 | 0.00 | 1.54-2.46 | 1.98 | 0.00 | 1.45-2.69 |
| Region of Origin |  | | |  |  |  |
| Middle east & north Africa | 1.61 | 0.00 | 1.50- 1.73 | 2.20 | 0.00 | 1.91-2.54 |
| South America | 0.46 | 0.07 | 0.19-1.08 | 1.33 | 0.78 | 0.18-9.98 |
| Sub-Saharan Africa | 0.53 | 0.00 | 0.45-0.61 | 0.68 | 0.10 | 0.42-1.08 |
| Australasia | 5.77 | 0.09 | 0.75-44.50 | 1.99 | 0.51 | 0.26-15.51 |
| North America & Europe | 1.66 | 0.00 | 1.23-2.22 | 0.95 | 0.88 | 0.48-1.86 |
